# Vancomycin Anaphylaxis and Kounis Syndrome Case Report

**DOI:** 10.7759/cureus.64740

**Published:** 2024-07-17

**Authors:** Brandon W Cunningham, Shreya Shah, Saptars Biswas

**Affiliations:** 1 Anesthesiology, Grand Strand Medical Center, Myrtle Beach, USA; 2 Internal Medicine, Edward Via College of Osteopathic Medicine-Carolinas, Spartanburg, USA; 3 Trauma and Acute Care Surgery, Grand Strand Medical Center, Myrtle Beach, USA

**Keywords:** cardiovascular collapse, hypersensitivity reaction, anaphylactoid reaction, anaphylaxis, vancomycin

## Abstract

Preoperative antibiotic administration has become a standard practice to mitigate the risk of surgical site infections; however, it is not devoid of potential complications. This report delves into the case of a 67-year-old male who underwent a routine preoperative vancomycin infusion prior to an elective total knee arthroplasty and subsequently experienced an unexpected adverse reaction including diaphoresis, cutaneous flushing, hypotension, and eventually unresponsiveness involving cardiovascular collapse and non-ST-segment elevation myocardial infarction (NSTEMI), despite minimal underlying coronary artery disease. The report focuses on the management of the emergent condition at our facility, highlighting the immediate response and following management of additional complications. The successful management led to a full recovery with the electrocardiogram (EKG) returning to the preoperative baseline of sinus rhythm with first-degree atrioventricular (AV) block within two hours of the event. This study contributes valuable insights into the complexities associated with preoperative antibiotic use, underscores the importance of understanding individual patient profiles, and raises awareness of potential risks and strategies to be used for antibiotic administration from a healthcare professional perspective.

## Introduction

Preoperative antibiotic prophylaxis is a critical component in reducing surgical site infections. The selection of antibiotics is tailored based on individual patient profiles, surgical procedures, and microflora involved. Commonly used antibiotics include first-generation cephalosporin (cefazolin) or second-generation cephalosporin (cefuroxime), which are more effectively used against a broader spectrum of gram-positive and gram-negative pathogens. However, for patients with a beta-lactam allergy, at a higher risk for MRSA infection, or based on the microflora involved, the glycopeptide antibiotic (vancomycin) is used [1]. While effective, the antibiotic vancomycin is known for its adverse reactions ranging from nephrotoxicity to infusion-related reactions, such as "red man syndrome." In animal studies, it has been shown that vancomycin is a direct stimulator of mast cell degranulation [2]. Vancomycin infusion reaction (VIR), formerly described as red man syndrome, is dependent upon the infusion rate, with resulting erythema and flushing of the skin that can develop across the upper body. In addition, like all compounds, there is the possibility of IgE-mediated anaphylactic type one hypersensitivity reactions and non-IgE-mediated anaphylactoid reactions, which occur due to direct chemical stimulation of mast cells [3,4]. This histamine release can result in vasoplegia, from which the associated hypotension can lead to decreased tissue perfusion and oxygenation, resulting in impaired myocardial function and eventual cardiac arrest [2,5-6]. In the perioperative setting when vancomycin may be used for surgical antibiotic prophylaxis, being able to ascertain an underlying cause of an abrupt, life-threatening event is paramount to providing effective and appropriate patient care.

## Case presentation

A 67-year-old, 112 kg male was scheduled to undergo elective total knee arthroplasty for osteoarthritis of the left knee with planned preoperative medications to include 1.75 g of intravenous (IV) vancomycin in 500 ml 0.9% normal saline solution, which was a weight-based dose and surgeon preference for surgical site infection prophylaxis in the setting of implantable hardware being used in the procedure. It was to be a slow infusion initiated preoperatively and continued intraoperatively until completed. The patient’s only reported allergy was to oxycodone, to which he experienced hives and had occurred over 20 years prior. Other past medical history was limited to hypertension and hyperlipidemia, as well as preoperative electrocardiogram (EKG) showing first-degree atrioventricular (AV) block, with no personal history of cardiac disease or antibiotic-resistant infections. Baseline vital signs were BP 104/61, HR 50 BPM, and oxygen saturation 99%.

Immediately upon connection of the primed IV line to the patient’s peripheral IV and the infusion initiated, the patient reported the sensation of fire spreading up his arm and began experiencing diaphoresis, cutaneous flushing, and hypotension, at which point a rapid response event was called, bringing in additional staff and expediting the patient being emergently brought to the post-anesthesia care unit after he shortly became unresponsive. At that time, the patient was found to have pulseless electrical activity (PEA) on cardiac monitor, and advanced cardiac life support (ACLS) management was begun including cardiopulmonary resuscitation, IV crystalloid fluid boluses, endotracheal intubation, arterial line and left femoral triple lumen central venous catheter placement with multiple doses of epinephrine and sodium bicarbonate, and initiation of a dopamine drip when the patient had a period of initial bradycardia during his hypotensive state. After roughly 10 minutes, a return of spontaneous circulation (ROSC) was achieved. Approximately 20 minutes later, the patient went into ventricular fibrillation, and ACLS management was restarted with defibrillation performed at 360 J, with 1 mg epinephrine, sodium bicarbonate, and amiodarone given. The dopamine drip was discontinued and transitioned to a norepinephrine drip. ROSC was again achieved after five minutes of CPR.

**Figure 1:**
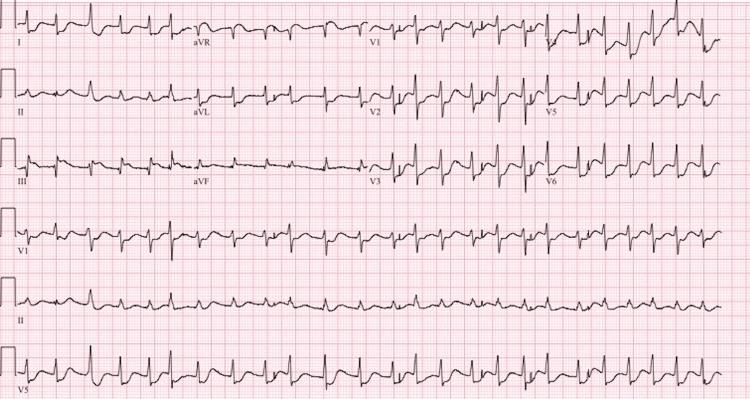
Initial electrocardiogram (EKG) showing atrial fibrillation with rapid ventricular response and ischemic changes after pulseless electrical activity (PEA) and ventricular fibrillation (Vfib) arrest.

**Figure 2:**
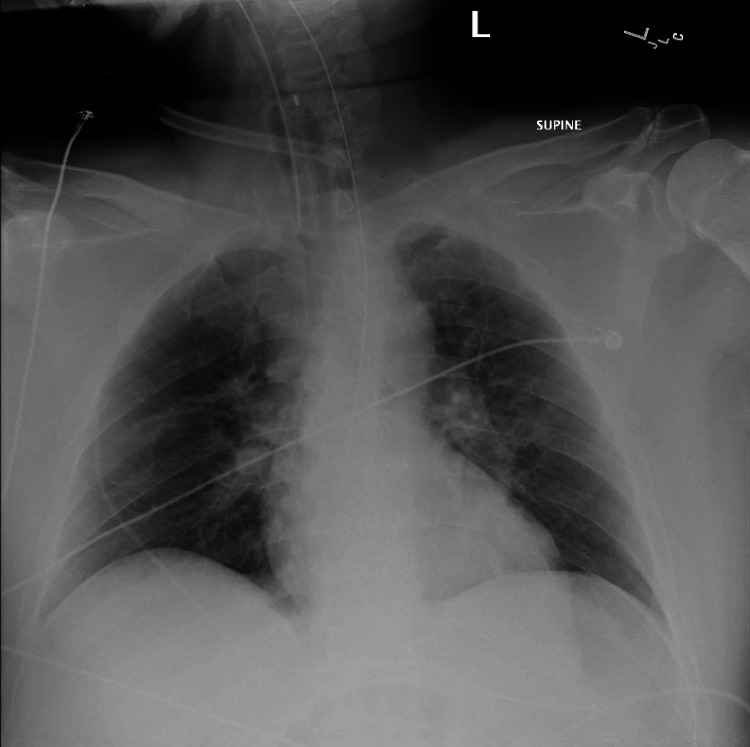
Post-event chest X-ray showing hilar fullness, likely from early pulmonary edema

A 12-lead EKG (Figure 1), chest X-ray (Figure 2), labs (Table 1), and cardiology consultation were obtained. The patient received IV diphenhydramine 25 mg q6hr, methylprednisolone 60 mg q8hr, famotidine 20 mg q12hr, and sedation with fentanyl and midazolam drips after ROSC had been obtained for a second time and the patient remained stable. Chest X-ray showed hilar fullness likely from early pulmonary edema. EKG at the time showed atrial fibrillation with rapid ventricular response with signs of ischemia including profound ST depressions and ST elevations seen in Lead III. Once hemodynamically stable, the patient was taken for emergent CT angiography to rule out pulmonary embolism or aortic dissection. Two hours after the event, the patient’s EKG returned to the preoperative baseline of first-degree AV block. Afterward, the patient was transported to the Medical Intensive Care Unit for observation and management by the medical critical care team that included correction of metabolic abnormalities. The patient was taken for cardiac catheterization with coronary angiography, which showed mild nonobstructive atherosclerotic disease in the LAD, LCx, and RCA with mild catheter-induced coronary spasm in the RCA. Transthoracic echocardiogram showed normal LVEF at 60-65% with no regional wall or valvular abnormalities.

**Table 1:** Laboratory trend following the event

Lab value	Time	Level	Facility reference range
Lactic acid	10:05	7.6	0.7-2.1 mmol/L
	12:30	7	
	16:05	2.6	
	19:37	2.4	
Troponin	10:05	<0.012	0-0.034 ng/mL
	12:30	0.474	
	01:58	3.34	
Potassium	09:55	4.8	3.5-5.1 mmol
	10:51	2.2	
	13:41	2.9	
ABG pH	09:55	7.285	7.35-7.45
	12:30	7.242	
	16:05	7.326	
Tryptase	10:05	33.9	2.2-13.2µg/L

No further events occurred overnight, and the patient successfully underwent spontaneous breathing trial and extubation the following morning after the reaction. Upon examination at the bedside 24 hours after the event, the patient was alert and oriented, being able to recollect the events and sensations leading up to his loss of consciousness. The patient was discharged home on hospital day three with no residual complications at that time. Three months after the event, the patient was able to undergo successful total knee arthroplasty without issue.

## Discussion

This patient had a major adverse reaction to the IV infusion of vancomycin, which resulted in major complications including cardiovascular collapse, non-ST-segment elevation myocardial infarction (NSTEMI), lactic acidosis, respiratory acidosis, and severe hypokalemia, along with rib and sternal fractures secondary to CPR. With the patient's history of mild coronary artery disease and elevated troponins during the event, one of the sequelae from the infusion is suspected to be vasospasm and demand ischemia. The event is complicated by the difficulty of discerning whether what occurred was VIR, anaphylaxis, or an anaphylactoid reaction.

All three of these situations are rare perioperative complications. Close monitoring of vitals prior to surgery, medical optimization, and perioperative measures are utilized to catch catastrophic complications such as these early. According to the National Anesthesia Clinical Outcomes Registry (NACOR) and National Surgical Quality Improvement Program (NSQIP), the incidence rates for cardiac arrest perioperatively are 1/1400 and 1/1800, respectively, with each one being less than 1% [7]. Perioperative anaphylaxis is attempted to be prevented through thorough documentation of the patient’s allergies, inclusion in surgical timeouts, and preventive measures to avoid providing known allergens to patients. However, even with proper management of known allergies, new onset reactions can make it difficult to identify a source in the perioperative setting with the amount of medications often provided to patients. The clinical presentation of anaphylaxis may not be initially evident and only appears as cardiac arrest. Rates for perioperative anaphylaxis are estimated to be approximately 15 cases per 10,000 [8]. The non-immune-mediated VIR and anaphylactoid reactions are minimized through gradual infusion. It was well documented and discussed that this patient received a slow vancomycin infusion rate, yet the experienced reaction was rapid. The rate of anaphylactic reaction to vancomycin is so low that they are not yet quantified in current literature. A common but not always necessary prerequisite for anaphylaxis is prior exposure and sensitization through IgE antibodies primed to a specific compound, which will allow for more rapid and exaggerated responses for subsequent exposures [3].

In this case, this patient was discovered to have received at least three vancomycin infusions previously for neurosurgical procedures. Tryptase is an important mediator released from mast cells during anaphylaxis, contributing to vascular changes, bronchoconstriction, and other associated symptoms during this type one hypersensitivity reaction. This patient had an elevated tryptase level, as seen in Table 1, which has been previously reported as being a differentiator supporting anaphylaxis as opposed to anaphylactoid and VIR [9].

To sum up the findings, this patient experienced an anaphylactic shock as suggested by hypotension, diaphoresis, flushing, and other hallmarks including elevated tryptase that resulted in widespread metabolic abnormalities and eventually cardiac arrest. More specifically, the association of this patient's anaphylactic shock and eventually cardiac arrest portrays a rare event called Kounis syndrome. Kounis syndrome, known as "allergic angina" or "allergic myocardial infarction," can occur in those with or without coronary artery disease who develop coronary artery spasms from a pathogenesis of mast cell activation-induced inflammatory cytokine release [10]. The inflammatory mediators in this pathogenesis can be triggered by food, insects, or allergies to certain medications. In addition, there are three variants of Kounis syndrome where type I deals with allergic vasospastic angina and myocardial infarction due to endothelial dysfunction along with normal troponins, type II can occur in those with underlying coronary artery disease where an allergic reaction leads to myocardial infarction and elevated troponins, and type III occurs in those with coronary thrombosis and presence of mast cells [10]. In this particular case, type II is presented based on the constellation of the patients' prior mild coronary artery disease, elevated troponins, and anaphylactic shock.

## Conclusions

After a review of the patient’s presentation and laboratory, imaging, and further workup findings, it was determined that he likely experienced an anaphylactic reaction to vancomycin, resulting in cardiovascular collapse and other complications. 

Differentiation of possible diagnoses of adverse reactions in the perioperative setting is important for the management of surgical patients who may be receiving both new and previously received medications. The patient's rapid onset of severe symptoms underscores the potential life-threatening nature of this adverse drug reaction and how prompt recognition and management are of critical importance. Airway securement, ventilatory support, and aggressive resuscitation measures are inherent to critical care and played a pivotal role in this patient's favorable outcome. However, further identification of causative factors is foundational for the prevention of future adverse events. The case described above is an effective example of how a major adverse reaction could be managed and worked up in the inpatient setting.
